# Human cognitive ability is influenced by genetic variation in components of postsynaptic signalling complexes assembled by NMDA receptors and MAGUK proteins

**DOI:** 10.1038/tp.2013.114

**Published:** 2014-01-07

**Authors:** W D Hill, G Davies, L N van de Lagemaat, A Christoforou, R E Marioni, C P D Fernandes, D C Liewald, M D R Croning, A Payton, L C A Craig, L J Whalley, M Horan, W Ollier, N K Hansell, M J Wright, N G Martin, G W Montgomery, V M Steen, S Le Hellard, T Espeseth, A J Lundervold, I Reinvang, J M Starr, N Pendleton, S G N Grant, T C Bates, I J Deary

**Affiliations:** 1 Centre for Cognitive Ageing and Cognitive Epidemiology, Department of Psychology, University of Edinburgh, Edinburgh, UK; 2 Medical Genetics Section, The University of Edinburgh Molecular Medicine Centre, Institute of Genetics and Molecular Medicine, Western General Hospital Edinburgh, Edinburgh, UK; 3 Genes to Cognition Programme, Centre for Clinical Brain Sciences and Centre for Neuroregeneration The University of Edinburgh, Edinburgh, UK; 4 Center for Medical Genetics and Molecular Medicine, Haukeland University Hospital, Bergen, Norway; 5 Dr E. Martens Research Group for Biological Psychiatry, Department of Clinical Science, University of Bergen, Bergen, Norway; 6 Queensland Brain Institute, The University of Queensland, Brisbane, QLD, Australia; 7 Centre for Integrated Genomic Medical Research, University of Manchester, Manchester, UK; 8 Public Health Nutrition Research Group Section of Population Health, University of Aberdeen, Aberdeen, UK; 9 Institute of Applied Health Sciences, University of Aberdeen, Aberdeen, UK; 10 Centre for Clinical and Cognitive Neurosciences, Institute Brain, Behaviour and Mental Health, University of Manchester, Manchester, UK; 11 Queensland Institute of Medical Research, Brisbane, QLD, Australia; 12 Department of Psychology, University of Oslo, Oslo, Norway; 13 KG Jebsen Centre for Psychosis Research, Oslo University Hospital, Oslo, Norway; 14 Department of Biological and Medical Psychology, University of Bergen, Bergen, Norway; 15 Kavli Research Centre for Aging and Dementia, Haraldplass Hospital, Bergen, Norway

**Keywords:** GWAS, intelligence, NMDA-RC, pathway analysis, synapse

## Abstract

Differences in general cognitive ability (intelligence) account for approximately half of the variation in any large battery of cognitive tests and are predictive of important life events including health. Genome-wide analyses of common single-nucleotide polymorphisms indicate that they jointly tag between a quarter and a half of the variance in intelligence. However, no single polymorphism has been reliably associated with variation in intelligence. It remains possible that these many small effects might be aggregated in networks of functionally linked genes. Here, we tested a network of 1461 genes in the postsynaptic density and associated complexes for an enriched association with intelligence. These were ascertained in 3511 individuals (the Cognitive Ageing Genetics in England and Scotland (CAGES) consortium) phenotyped for general cognitive ability, fluid cognitive ability, crystallised cognitive ability, memory and speed of processing. By analysing the results of a genome wide association study (GWAS) using Gene Set Enrichment Analysis, a significant enrichment was found for fluid cognitive ability for the proteins found in the complexes of *N*-methyl-D-aspartate receptor complex; *P*=0.002. Replication was sought in two additional cohorts (*N*=670 and 2062). A meta-analytic *P*-value of 0.003 was found when these were combined with the CAGES consortium. The results suggest that genetic variation in the macromolecular machines formed by membrane-associated guanylate kinase (MAGUK) scaffold proteins and their interaction partners contributes to variation in intelligence.

## Introduction

Performances on diverse cognitive tasks are universally positively correlated and a latent trait of general cognitive ability (intelligence) can be quantified, typically accounting for just under half of the variation in any large battery of cognitive tests.^1^ This trait is stable and predictive of health, longevity and a range of socioeconomic outcomes.^2^ Genome-wide analyses of common single-nucleotide polymorphisms (SNPs) indicate that, over the life course, these SNPs or variants in linkage disequilibrium (LD) with these SNPs jointly explain between 26 and 51% of the variance in intelligence differences.^3,4^ Despite this, no single polymorphism has been reliably associated with general intelligence.^5^ Functional networks of genes that jointly regulate a complex function^6^ may allow aggregation of information present in current SNP chips to elucidate the molecular pathways underlying cognitive differences.^7^ Here, we combine gene-based statistics (Versatile Gene-based Association Study, VEGAS)^8^ with a competitive test of enrichment (Gene Set Enrichment Analysis, GSEA)^9,10^ to test whether genetic variation in the postsynaptic protein complexes of the excitatory synapses in the human brain show a greater association with intelligence than genes from outside these networks. Testing for associations between cognitive abilities and gene networks might yield a substantial increase in power compared with single-gene methods.^11^

Candidate phenotypes implicated in cognitive differences centre on the central nervous system including variation in white matter integrity^12,13^ and brain volume.^14,15^ However, to explore the genetic foundations of intelligence further, a more specific target is preferable. The synapse is a particularly rich target system both because of the large number of genes expressed^16^ and because of direct evidence for the effects of mutations in this system on cognition.^17^ Here we investigate a specific component within the synapse, the postsynaptic density (PSD).

Mutations in genes expressed in the PSD have been linked to many dozens of neurological and cognitive disorders.^18, 19, 20^ The PSD can update its own responsiveness to subsequent input on very short and long time scales.^21^ At the genetic level, evidence suggests that the elaboration of complex learning involved duplication and subsequent divergence of genes in the PSD.^22^ This was followed by strong conservation of function in the vertebrate line,^23^ indicative of a finely tuned system. The PSD, therefore, is a promising candidate for seeking genes in which variation is associated with intelligence.

### The PSD and associated complexes

**Figure 1:**
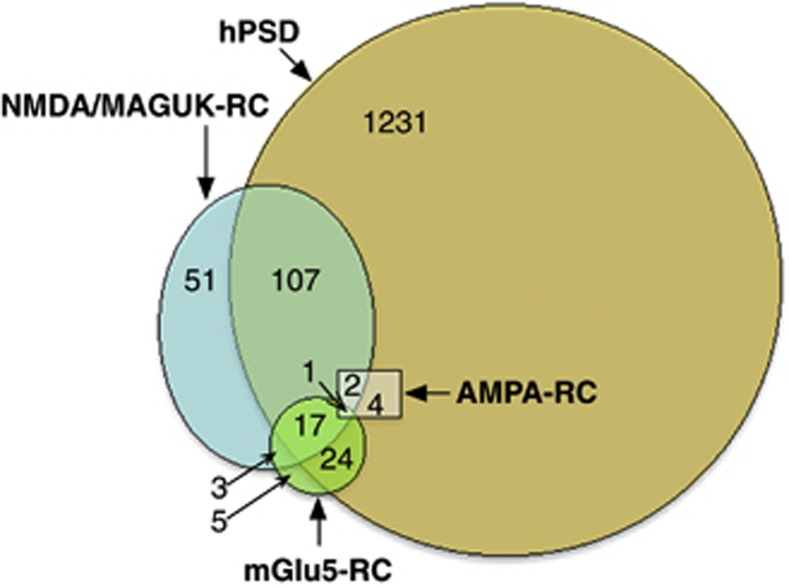
Venn diagram showing the overlap of three gene complexes and their relative genetic overlap within the proteins of the full human postsynaptic density (hPSD). Numbers of genes in each gene set and overlap of these are also shown. Note: The full hPSD consists of all genes associated with proteins in the hPSD.^18^ The genetic constituents of the AMPA-RC (α-amino-3-hydroxy-5-methyl-4-isoxazoiepropionic acid receptor complex), mGlu5-RC (metabotropic glutamate 5 receptor complex) and NMDA-RC (*N-*methyl-D-aspartate receptor complex) are taken from mouse-based proteomic experiments.^19^

**Figure 2:**
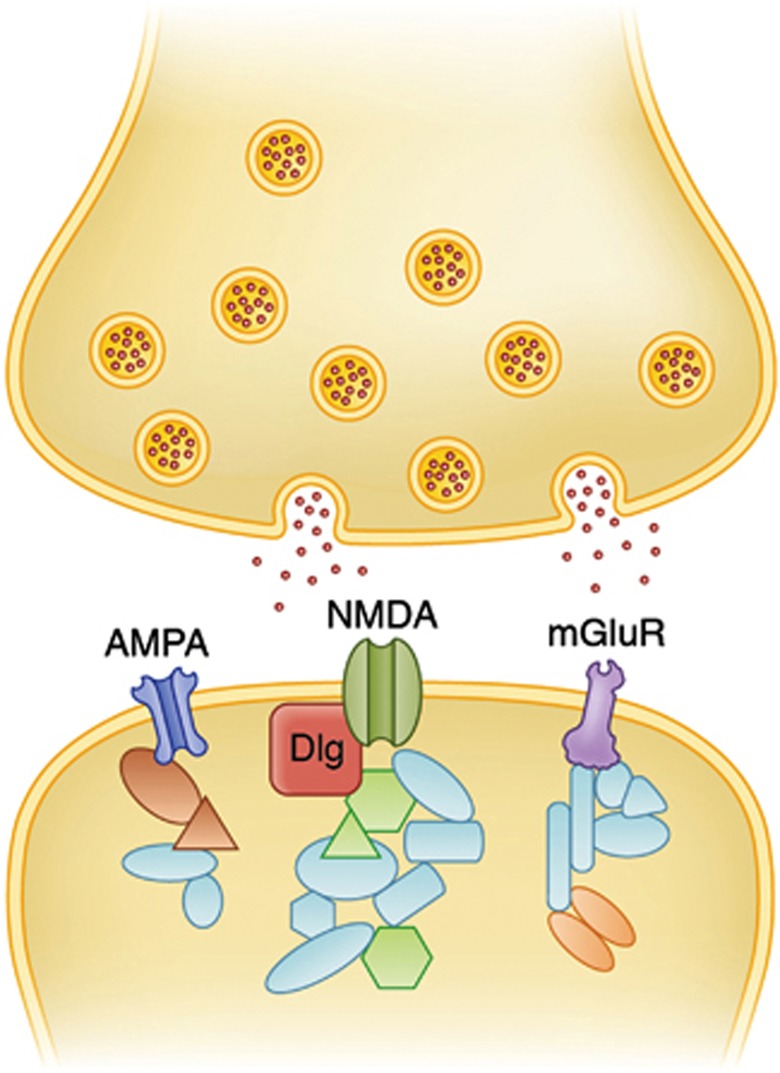
Schematic of a central nervous system excitatory synapse showing the proteins in the postsynaptic terminal organised into multi protein complexes assembled with glutamate receptors (AMPA-RC (α-amino-3-hydroxy-5-methyl-4-isoxazoiepropionic acid receptor complex), NMDA (*N*-methyl-D-aspartate and mGluR (metabotropic glutamate 5 receptor complex) receptors shown).

Among the proteins comprising the mammalian PSD, three complexes are of particular importance in mediating neural transmission: The NMDA-RC (*N*-methyl-D-aspartate receptor complex), mGlu5-RC (the metabotropic glutamate 5 receptor complex) and the AMPA-RC (α-amino-3-hydroxy-5-methyl-4-isoxazolepropionic acid receptor complex)^19^ (see Figures 1 and 2).

The AMPA-RC is the primary basis of rapid excitatory neurotransmission in the mammalian brain^24,25^; in addition, the induction of long-term potentiation (LTP) is induced, in part, by the summation of AMPA-mediated excitatory postsynaptic potentials.^26^ Using *in vivo* rat models, it has been possible to show that an increase in the amplitude and duration of the excitatory postsynaptic potentials, produced by AMPA-RC activation, is associated with an increase in LTP and performance in memory tasks.^27^

Synaptic plasticity is dependent on both the NMDA-RC^28^ and mGlu5-RC.^29^ The mGlu5-RC, consisting of some 52 proteins forming the metabotropic Gα_q_-linked G-protein-coupled glutamate receptor,^19^ is closely associated with longer-term modulation and maintenance of LTP.^30, 31, 32, 33, 34^ NMDA/MAGUK-RC is involved in rapid processing of information and updating of AMPA-RC responsiveness.^28^ The NMDA-RC consists of neurotransmitter receptors, ion channels and signalling proteins scaffolded at the postsynaptic membrane where they function to convert information in patterns of action potentials into biochemical signals underlying memory and other aspects of cognition.^35^ Mutations in NMDA-RC have been implicated in the aetiology of over 100 brain disorders, including those with cognitive deficits such as schizophrenia, autism and intellectual disability;^18,20,35, 36, 37^ this supports the linkage of the NMDA-RC to both cognitive and psychiatric disorders. During the review process, an additional synaptic component, the activity-regulated cytoskeleton-associated (ARC) protein, was included. ARC has been reliably associated with both LTP^38^ and LTD^39^ with ARC mRNA being transported to active synaptic regions via the dendritic spine where it is then translated and serves to modulate AMPA trafficking.^40^ *De novo* mutations in the ARC protein have been implicated in schizophrenia,^20^ a disease in part predicted by a low premorbid cognitive ability,^41^ which may be due to a shared genetic component between the two traits.^42^

Here we tested for an association between genetic variation in these four gene networks and non-pathological cognitive variation. This was done using experimentally determined gene sets based on proteins detected in the PSD of human and mouse brains.^18,19^ The cognitive phenotypes studied were general cognitive ability, fluid cognitive ability, crystallised cognitive ability, memory and processing speed. The GSEA^9,10^ program was used to test whether gene sets corresponding to these components showed significant enrichment for the five cognitive phenotypes. The discovery samples were those of the Cognitive Ageing Genetics in England and Scotland (CAGES) consortium.^3^ Replication of significant findings was sought in two independent samples from Norway and Australia.

## Materials and methods

### Participants

The CAGES consortium, consisting of 3511 relatively healthy middle-aged and older individuals, includes the Lothian Birth Cohorts of 1921 and of 1936 (LBC1921 and LBC1936),^43^ the Aberdeen Cohort of 1936 (ABC1936)^44^ and the Manchester and Newcastle Longitudinal Studies of Cognitive Ageing Cohorts.^45^

The LBC1921 cohort consists of 550 (316 females) individuals, most of whom took part in the Scottish Mental Survey 1932.^46, 47, 48^ Most resided in Edinburgh city and the surrounding Lothian region at about age 79 when they were first recruited to the LBC1921 study between 1999 and 2001. Their mean age was 79.1 years (s.d.=0.6). Subjects were identified by examining the records of those registered with a general practitioner in the area and by media advertisements. They were healthy, older individuals all of whom lived independently in the community.^47^ Venous whole blood was collected for DNA extraction following informed consent. Ethical approval was granted by The Lothian Research Ethics Committee.

LBC1936 was recruited in a similar manner to LBC1921. It consists of 1091 (543 females) individuals most of whom took part in the Scottish Mental Survey 1947. Most were living in and around Edinburgh when they were recruited to the LBC1936 between 2004 and 2007. Their mean age was 69.5 years (s.d.=0.8).^49^ They were healthy, older individuals all of which lived in the community. Venous whole blood was collected for DNA extraction following informed consent. Ethical approval was granted by Scotland's Multicentre Research Ethics Committee and the Lothian Research Ethics Committee.

ABC1936 consists of 498 (255 females) individuals who were drawn from the original members of Scottish Mental Survey 1947 and were living in the Aberdeen area when recruited between 1999 and 2003. Their mean age was 64.6 (s.d.=0.9) years. They were healthy, older individuals all of whom lived independently in the community.^44^ Each had venous whole blood extracted in order to collect DNA samples following informed consent. The Grampian Research Ethics Committee granted ethical approval.

The Manchester and Newcastle Longitudinal study of Cognitive Ageing Cohorts were assembled in order to measure individual differences in the effects of ageing on mental ability.^45^ Participants were collected and tested over a 20-year period beginning in 1983/1984 that resulted in an initial sample size of 6063 (4238 females) with a median age of 65 years ranging from 44 to 93 years. Participants were healthy and lived independently in the community.^45^ Venous whole blood was taken for DNA extraction from 805 of the Manchester cohort (572 females) and 758 of the Newcastle cohort (536 females) following informed consent. Ethical approval was granted by the University of Manchester.

The first replication cohort was formed from healthy twins and their non-twin siblings recruited as part of the Brisbane Adolescent Twin Study (BATS)^50^ and those who subsequently had cognitive phenotypes collected through participation in cognition and imaging studies (*n*=2062).^51,52^ Together they were drawn from 928 families that included 339 monozygotic pairs and one set of monozygotic triplets. Participants were female (1093) and male (969), with ages ranging from 15.4 to 29.6 years (mean=16.6, s.d.=1.5). The studies were approved by the Human Research Ethics Committee at the Queensland Institute of Medical Research, as well as the institutional ethics boards at the University of Queensland and the Wesley Hospital.

The second replication cohort was the Norwegian Cognitive NeuroGenetics (NCNG) cohort^53^ that consists of 670 healthy individuals (457 females) with an age range of 18–79 years (mean=47.6, s.d.=18.3). Participants were drawn from and tested in Bergen (*n*=171) and Oslo (*n*=499). Permission to take and store blood samples for genotyping along with cognitive and magnetic resonance imaging data in a bio-bank and to establish a registry for relevant information was granted by the Norwegian Department of Health. Ethical approval was granted by the REK Sørøst (Norwegian Ethical Committee), NCNG: project ID S-03116.

### Cognitive phenotypes

Four cognitive phenotypes were tested for association in this study. These were general fluid cognitive ability (*gf*), crystallised cognitive ability, memory and processing speed. Fluid ability describes an individual's ability to deal with novel information,^54^ often involving abstract reasoning tasks with little or no verbal component. Whereas different tests were used in the construction of each general factor, correlations between *g* factors formed from different batteries are typically high.^55^

The *gf* score for the three Scottish cohorts was derived by using the raw scores from each test and subjecting them to a principal component analysis where the first unrotated component was extracted using regression. Following this, the effects of age and sex were controlled using a linear regression model with the factor score being the dependent variable. The standardised residuals were extracted from this model and were used in subsequent analyses.

For the LBC1921 cohort, *gf* was derived from the Moray House Test,^46^ Raven's Standard Progressive Matrices,^56^ phonemic verbal fluency^57^ and Wechsler Logical Memory scores^58^. The general factor for LBC1936 was formed from six non-verbal tests from the Wechsler Adult Intelligence Scale III^UK^ (WAIS-III^UK^): Digit Symbol Coding, Block Design, Matrix Reasoning, Digit Span Backwards, Symbol Search, and Letter-number Sequencing^59^. The general fluid ability factor for ABC1936 was formed from the Rey Auditory Verbal Learning Test,^57^ the Uses of Common Objects,^60^ Raven's Standard Progressive Matrices^56^ and Digit Symbol from the WAIS Revised (WAIS-R).^61^

The factor for general fluid ability in the Manchester and Newcastle ageing cohort was derived using the two parts of the Alice Heim test 4^62^and the four sub-tests of the Culture Fair Test.^63^ Age at test and sex were controlled using residualisation, and these standardised residuals for each of the tests were then subjected to a maximum likelihood factor analysis. A general factor was extracted using regression, and missing data points were accounted for by sampling the posterior distribution of factor scores for each subject using Mplus.^64^

Crystallised ability describes the level of knowledge an individual has acquired over the life course.^54^ It is typically assessed by means of language-based tests including reading ability or measurements of vocabulary. For LBC1921, LBC1936 and ABC1936 this was represented by the score from the National Adult Reading Test.^65^ For the Manchester and Newcastle cohorts, sections A and B from the Mill Hill vocabulary test^66^ were used. These sections were administered without a time limit and were summed to give a single score. The raw scores from each of the tests representing crystallised ability were subjected to a linear regression with age and sex as predictors and the test score as the dependent variable. The standardised residuals from these models were used for all subsequent analyses.

Verbal declarative memory (memory) and information processing speed (speed) were each measured by a single test in each cohort. In the LBC1921 cohort, the total score from both the immediate and delayed recall sections of the Logical Memory test from the Wechsler memory scale revised^58^ was used. In LBC1936, it was the total from the immediate and delayed recall sections from the logical memory test from WAIS-III^UK^.^59^ In ABC1936, a modified version of the Rey Auditory and Verbal Learning Test^57^ was used where a set of 15 words was read to the subject who then repeated aloud as many as they could. Following this, the same list was read out again and the subject was again asked to recall as many words as they could. The word list was presented a total of five times and the participants final score was the total number of words summed across the five presentations. In the Manchester and Newcastle cohorts, a cumulative verbal recall task^45,55^ was used in which four presentations of a list of 15, six letter nouns was read aloud to the participant. A recall phase was administered between each presentation where the participants were instructed to write down as many of the words as they could recall. The final score was the total recalled across all four presentations. The raw scores from each of the tests representing memory were subjected to a linear regression with age and sex as predictors, and the test score as the dependent variable. The standardised residuals from these models were used for all subsequent analyses.

Information processing speed (speed) was measured in each cohort using a single test. The digit symbol subtest of the WAIS-III^UK^ ^59^ was carried out by LBC1921 and LBC1936, whereas in ABC1936 the WAIS-R version^61^ was used. The Savage Alphabet Coding Task^67^ was used in the Manchester and Newcastle cohorts. The raw scores from each of the single tests representing speed were subjected to a linear regression with age and sex as predictors, and the test score as the dependent variable. The standardised residuals from these models were used for all subsequent analyses. In response to a reviewer's request, a fifth cognitive phenotype, a general factor of cognitive ability (*g*), was created and tested. This *g* factor was constructed using the tests measuring fluid and crystallised abilities in each of the cohorts in the CAGES consortium. A separate *g* factor was derived within each cohort. In ABC1936, LBC1921 and LBC1936 the total number of correct responses on the National Adult Reading Test was included along with the tests used in the respective *gf* phenotypes in principal component analysis. The participants' scores on the first unrotated component were extracted using regression. Following this, the effects of age and sex were regressed out.

In the Manchester and Newcastle cohorts, the effects of age and sex were regressed out from both the *gf* factor and the score from the Mill Hill vocabulary test. Following this the standardised residuals from the Mill Hill and the *gf* factor were summed and the mean derived. This mean score was used to represent the *g* factor.

### Replication cohorts

In the Australian Sample, performance IQ was used as an indicator of *gf*. This was derived from scores on the Spatial and Object Assembly tests according to the manual for the Multidimensional Aptitude Battery.^68^ Each test was administered with a 7-min time limit. A general factor of cognitive ability (*g*) was represented by the full-scale IQ score derived using the Multidimensional Aptitude Battery.^68^ In the Norwegian sample, the Matrix Reasoning subtest from the Wechsler Abbreviated Scale of Intelligence^69^ was used as an indicator of *gf*. Each participant's raw score from this test was subjected to a linear regression using their age and sex as predictor variables. The standardised residuals from this model were used in subsequent analyses.

### Genotyping and quality control

Genotyping and quality control procedures implemented here have been described previously;^3^ however, this study makes use of imputed data as detailed below. The 3782 participants in the discovery cohorts had DNA extracted and were genotyped for 599 011 common SNPs using an Illumina610 QuadV1 chip (Illumina, San Diego, CA, USA). After quality control, 549 692 SNPs were retained in 3511 participants (2115 females). Individuals were removed due to unresolved gender discrepancies, relatedness or call rate<0.95, as well as evidence of non-Caucasian descent. SNPs included in the analysis had a call rate of >0.98, minor allele frequency of >0.01 and a Hardy–Weinberg equilibrium test of *P*>0.001. Multidimensional scaling (MDS) analysis was performed to test for population stratification and any outliers were excluded. The first four MDS components, based on the remaining individuals, were then included as covariates in subsequent analyses.^3^ Imputation was performed in each cohort using the MACH^70^ software (v1.0.16) to the HapMap phase II CEU (NCBI build 36 release 22) reference panel. Imputed SNPs were retained for analysis with an imputation quality score of greater than 0.3 and a minor allele frequency of >0.005.

The genotyping and quality control for BATS have been described previously.^71^ In this Australian sample, 2104 participants had DNA extracted from blood and were genotyped on the Illumina Human 610-Quad chip (Illumina). Following quality control, 529 379 SNPs were retained in 2062 (1093 female and 969 male) participants. Individuals were removed due to unresolved gender discrepancies or evidence of non-Caucasian descent. SNPs were included if they met the criteria of call rate >0.95, minor allele frequency >0.01 and a Hardy–Weinberg equilibrium test of *P*>0.00001.^71^ Multidimensional scaling analysis of SNP data showed three components. To control for population stratification, all three components were entered as covariates along with age and sex in the analyses.

The genotyping and quality control for the NCNG have been described previously.^3,53^ For this Norwegian sample, DNA was extracted from blood using the Qiagen Gentra Autopure LS system (Qiagen, Valencia, CA, USA). Genotyping took place on the Illumina Human 610-Quad Beadchip (Illumina). Quality control was carried out using the ‘check.marker' function from the R package GenABEL.^72^ Identity-by-state was used to assess cryptic relatedness, with cases where Identity-by-state threshold exceeded 0.85 being removed. Population structure was assessed using multidimensional scaling analysis where individuals who were suspected of possible recent non-Norwegian ancestry were removed. Individuals were also removed if the heterozygosity value was >2 s.d. from the sample mean or where sex could not be determined. SNPs were excluded if the call rate was <0.95, a minor allele frequency of <0.01 and a Hardy-Weinberg Equilibrium (exact test) *P*-value<0.001. The final sample consisted of 554 225 SNPs in 670 individuals.

### PSD gene sets

The genes responsible for the expression of the PSD and its subcomponents are available at the G2C database (http://www.genes2cognition.org/db/GeneList). The size of the gene sets used along with the degree of overlap between the gene sets is shown in Figure 1.

The human-derived PSD (hPSD) was ascertained based on experimentally identified proteins, where hPSDs were isolated from neocortical samples of nine adults (mean age=47.0 years, s.d.=15.74, three females) who had undergone a variety of medically necessary neurosurgical procedures.^18^ The protein preparations were pooled into three samples from three individuals, each sample containing normal non-diseased tissue from at least two of three cortical regions (frontal, parietal and temporal lobes). These three samples were then subjected to proteomic profiling using liquid chromatography tandem mass spectrometry.^18^ The full set consisting of 1461 genes, details genes whose proteins were found in at least two pooled samples, whereas the consensus set features the 748 genes found in all three samples. Only autosomal genes were included in the present analyses leaving 1386 genes in the full hPSD and 714 in the concensus hPSD (94.8% of the full hPSD and 95.4% of the consensus list).^18^

The NMDA-RC gene set was based on previous studies.^19^ NMDA-R complexes were isolated using affinity to a peptide derived from the carboxy terminus of the NR2B protein and analysed by liquid chromatography tandem mass spectrometry. The identified list of proteins overlapped substantially with an NMDA receptor complex (NRSC) identified earlier.^73^ The earlier complex was an amalgamation of lists derived by immunoprecipitation from mouse forebrain with an NMDA receptor NR1 subunit antibody and the same NR2B carboxy terminal peptide. The combined NMDA-RC list consists of 186 genes of which 181 are autosomal and were included in this study. Genes coding for the mGlu5-RC were those identified using an antibody against mGluR5 protein in rat brain lysates.^74^ Of 52 mouse orthologues of these genes that have been identified,^19^ all 50 autosomal genes were included in the present analyses. The AMPA-RC comprised a set of nine proteins and corresponding genes isolated by immunoprecipitation using an antibody against the GluR2 protein (*Gria2*).^19^ The seven autosomal genes from this set were included in the present analyses. The ARC protein gene set was taken from Kirov *et al*^20^ and included the same 25 of 28 (89.3%) genes used in their analyses.

### Statistical analysis

**Figure 3:**
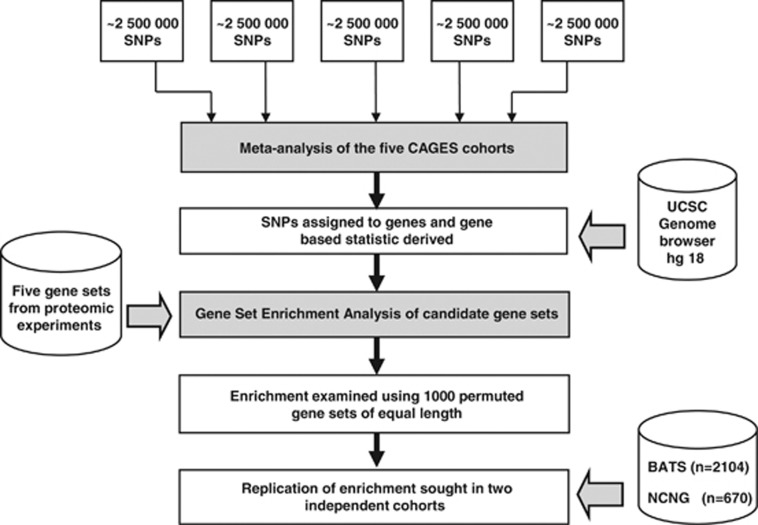
Data processing stages from top to bottom. The five cohorts from the Cognitive Ageing Genetics in England and Scotland (CAGES) consortium underwent single-marker analysis^70^ separately before the results were meta-analysed.^75^ Single-nucleotide polymorphisms (SNPs) were then assigned to genes based on their position as indicated in the UCSC Genome browser hg18 assembly and a gene-based statistic was derived using Versatile Gene-Based Association Studies (VEGAS).^8^ *A priori-*selected gene sets detailing the molecular composition of the PSD were brought in^18,19^ and enrichment of these sets in cognition was sought using Gene Set Enrichment Analysis (GSEA).^9,10^ Gene sets which were enriched were then compared with 1000 randomly selected gene sets of the same length to examine the strength of the enrichment found. Gene sets which survived this procedure were then taken forward for replication in two independent cohorts.

Data were processed through the following six steps (see Figure 3). First, association analyses were performed in each cohort using Mach2QTL.^70^ Second, these results were then combined using an inverse variance-weighted meta-analysis in METAL.^75^ The third step was to use VEGAS^8^ to conduct gene-based analyses of association for each of the five cognitive phenotypes on the results of the meta-analysis. SNPs were assigned to genes according to their position on the UCSC Genome browser hg18 assembly with a gene boundary of ±50 kb of 5′ and 3′ untranslated regions. The gene-based statistic was then derived using each SNP within the specified boundary, with VEGAS controlling for the number of SNPs in each gene and the LD between those SNPs. Gene-based *P*-values were then –log(10) transformed and rank ordered for each phenotype. In the fourth step, the specific gene set enrichment hypotheses were tested using a competitive test of enrichment, GSEA.^9,10^ GSEA uses a candidate list of gene identifiers and a genome-wide set of genes that are ranked by the strength of their association with a phenotype. GSEA tests whether gene identifiers in the candidate set fall higher in the genome-wide ranking than would be expected by chance. A running–sum Kolmogorov–Smirnov statistic weighted by the *P*-value from the genome-wide gene ranking set is derived. This process is repeated and the final enrichment *P*-value corresponds to the proportion of runs in which the test gene set ranked higher than the permuted set. Here 15 000 permutations were used. Gene sets meeting the pre-determined discovery criteria of an uncorrected enrichment *P*-value ≤0.05, and/or false discovery rate (*FDR)*-corrected *q-*value of <0.25 were empirically validated as follows (step five). Each significant gene set (NMDA-RC and mGlu5-RC) was compared against *P*- and *FDR* values derived from 1000 randomly sampled gene sets of the same length.^76^ Empirical significance was set for *P-* and *FDR* values of the observed gene set as being smaller than 95% of those obtained in the random gene lists. Gene sets passing this criterion were taken forward to step six: replication in the BATS and NCNG cohorts.

### Replication

In the Australian BATS sample, initial analysis of the genotyped data was conducted using Multipoint engine for rapid likelihood inference (MERLIN),^77^ allowing control for relatedness between participants in this family-based sample. In the Norwegian NCNG cohort, single-marker analysis was carried out using PLINK.^78^ In both samples, an additive inheritance model was used and the same data processing steps were used as in the discovery cohort. As only the NMDA-RC gene set met the criteria to be deemed significant against any cognitive variable, it was the only set in which a replication was sought. Following replication, the enrichment *P*-values from each of the three cohorts (CAGES, NCNG and BATS) were combined using Stouffer's weighted Z-transform method.^79,80^ The discovery cohort *P*-value was corrected for multiple comparisons using a Bonferroni correction for the five gene sets tested × four phenotypes, that is, a correction for 20 tests (0.002 × 20=0.04) before being combined with NCNG and BATS.

## Results

Genome-wide association (GWA) analyses of the association between each of the five cognitive phenotypes was undertaken for the full set of imputed SNPs in each of the five CAGES cohorts. Analyses have already been reported for fluid and crystallised ability on non-imputed data;^3^ however, here we use imputed data. Moreover, we report for the first time the GWAS analyses for memory and processing speed phenotypes in these cohorts. A meta-analysis was then performed on results from the five cohorts using METAL.^75^ No SNP reached genome-wide significance for any of the five cognitive phenotypes.

### Gene-based association

Gene-based analysis of the meta-analytic SNP association data combining information from the five cohorts found no single gene significantly associated with any of the five phenotypes. The most significant gene-based *P*-values for general cognitive ability, fluid cognitive ability, crystallised ability, memory and processing speed, respectively, were for *FNBP1L* (*P*=3 × 10^−^^5^), *BCAR3* (*P*=4.0 × 10^−6^), *RFFL* (*P*=7.0 × 10^−^^5^), *OR4P4* (*P*=4.0 × 10^−^^5^) and *EIF5A2*, (*P*=4.9 × 10^−^^5^). The gene with most evidence for association in the earlier GWA in this cohort (*FNBP1L* for *gf*)^3^ ranked second in these analyses (*P*=1.9 × 10^−5^). This slight difference is likely to be because of the use of imputed SNPs in the present analyses, and that phenotype construction differed for the Manchester and Newcastle cohorts between this and the previous analysis.

### Enrichment analysis of PSD gene sets

Next we test our principal hypothesis that variation in genes that code for the proteins in the PSD is involved in the normal range of variation of cognitive abilities. GSEA analyses were performed on each the six gene sets for each of the cognitive phenotypes. Of the six gene sets, the NMDA-RC was significant (*P*=0.002) for *gf* (Table 1). mGlu5-RC had an *FDR* also under 0.25, but had a *P*-value of 0.133. The NMDA-RC was also found to have an enriched association with general cognitive ability (*P*=0.0084). No significant support for enrichment was found for any of the other three phenotypes for any other gene set. By comparison with 1000 randomly ascertained sets of 181 genes, both the *P*-value and *FDR* obtained for the NMDA-RC was lower than that of 99.7% of the random gene sets in the *gf* phenotype. In the case of the association of mGlu5-RC with *gf*, comparison with 1000 randomly sampled lists did not provide significant support for enriched association (observed *P*-value <83.0% *FDR* <84.1% of random gene sets). Upon examination, no significant enrichment was found between the ARC gene set and *gf*, crystallised ability, memory and mental speed with *P*-values of 0.87, 0.09, 0.61 and 0.68 being found, respectively.

To ensure that the enriched association was not driven by a single gene, the most significant gene from the NMDA-RC set and the mGlu5-RC set were removed. Once *DNM2* was removed from the mGlu5-RC list, no significant evidence of enrichment with *gf* remained. However, removing the top gene from the NMDA-RC gene set – *PRDX2 –*attenuated the enrichment with *gf* but it remained significant (*P*=0.006). This was repeated with *g,* where once the most significant gene was removed from the NMDA-RC (*PLCG1*), significance remained (*P*=0.024). These results support the hypothesis that genetic variation in NMDA-RC is associated with general intelligence differences and more specifically with fluid ability but not with the PSD, more broadly, nor the AMPA or mGlu5 receptor complexes.

### Replication

The enrichment of the NMDA-RC gene set in fluid cognitive ability was tested for replication in the Norwegian and Australian cohorts using identical methods to those used above in the discovery sample, that is, gene-based analysis using VEGAS, followed by a GSEA unit-weighted analysis with 15 000 permutations. Enrichment testing in the BATS and the smaller NCNG cohorts yielded, *P-*values of 0.012 and 0.371, respectively. The association remained significant in BATS after removing the top gene (*RAB3A*) from the set (*P*=0.024), indicating that multiple genes were contributing to the enrichment signal in both CAGES and BATS. A meta-analysis of these results for the NMDA-RC across the discovery cohort, and two replication samples was determined using Stouffer's-weighted Z-transform method.^79,80^ The probability of obtaining these results across the three independent cohorts, corrected for multiple testing in the discovery cohort and tested against the null hypothesis of no association was *P*=0.003. By omitting the discovery cohort, the enrichment of the NMDA-RC across BATS and NCNG remained significant (*P*=0.018), supporting the enriched association of the NMDA-RC with fluid ability. The NMDA-RC also demonstrated an enriched association with a general factor of cognitive ability in the BATS cohort *P*=0.043.

## Discussion

The present study used a hypothesis-driven approach to test the joint effect of multiple variants clustered in the same biological network on human intelligence differences. In drawing upon the synapse proteomic data sets, the results suggested that SNP variation in the genes encoding the NMDA/MAGUK receptor complex is enriched for association with both general cognitive ability and general fluid cognitive ability in humans. This finding linking NMDA-RC to fluid ability provides evidence that genetic variation in the macromolecular machines formed by MAGUK scaffold proteins and their interaction partners contributes to variation in intelligence.

By contrast with the NMDA-RC, other components of the PSD were not found to be significantly enriched for variation in cognitive abilities in this study. These results raise the question of why the NMDA-RC should be preferentially involved in fluid-type intelligence. The present results suggest that association of the NMDA-RC with *gf* does not simply follow from its being a part of the synapse or having a role in the excitatory transmission system, as three other systems found in the synapse did not show enrichment, and all are activated once the receptors bind with glutamate or are found only at glutamatergic synapses. However, the lack of an enriched association for the AMPA-RC or the mGlu5-RC could be due to the small numbers of genes involved in their expression, meaning that even greater sample sizes would be required to detect an enrichment of these complexes. The lack of an enriched association of ARC with *gf* may also reflect simply a lack of power. Alternatively, it may be that lack of enrichment with the ARC protein for cognition implies that this system is specific for schizophrenia rather than for general cognitive ability.

The NMDA-RC is enriched for both learning and synaptic plasticity phenotypes in mice,^35^ and the same proteins have been shown to be involved in human learning disabilities.^35^ These findings validate the utility of rodent models for human cognitive function. In addition, they suggest that combinations of SNPs in LD with common SNPs found within the genes of the NMDA-RC may result in variation in synaptic plasticity, which in turn is responsible for some of the observed differences in human intelligence.

Variation in the NMDA-RC has been implicated in schizophrenia^20,81^ and intellectual disability^17,37,82,83^ with mutations in individual scaffolding molecules SAP102/Dlg2 and PSD93/Dlg3 linked to these disorders, respectively. The present finding of a link between intellectual function and variation in the NMDA-RC therefore supports a genetic link between schizophrenia and intelligence, in keeping with behaviour genetic^42^ research, and also with recent polygenic risk studies of a sub-set of the present cohorts that indicated an overlap of polygenic risk factors for schizophrenia and for cognitive ageing.^84^ The genetic link between schizophrenia and cognitive abilities appears to be region rather than variant specific. Where *de novo* copy number variation at the NMDA-RC is associated with schizophrenia,^20^ it is common SNP variation, in the same region, which shows an enriched association with the normal range of cognitive abilities. However, neither the common SNPs nor copy number variations associated with schizophrenia have been shown to be associated with intelligence differences in a non-elderly cohort.^85^

Enrichment was found for fluid ability and not for crystallised ability, memory or processing speed. If gene effects directly impact on specific functions (rather than on general ability *per se*), then analyses targeting these specific functions (such as speed or memory) are known to be significantly more powerful than are analyses of a composite or latent factor such as fluid ability.^86^ Here, the enriched association of the NMDA-RC was found for the fluid ability composite rather than specific functions. The finding that genetic association for the fluid ability phenotype proved the stronger indicator, then, is compatible with generalist genetic action as opposed to functional specificity.^87^ This is further supported with the finding that the NMDA-RC is enriched for general cognitive ability. This enrichment was, however, attenuated compared with *gf,* indicating that genetic variation of the NMDA-RC is preferentially linked to non-verbal cognitive tasks and solving problems that incorporate novel information.

Whereas the mGlu5-RC gene set showed weak evidence of enrichment in the initial GSEA analysis, this did not survive permutation testing. It was shown to be due to a single gene, *DNM2*, rather than an over representation of mGlu5-RC genes in the upper portion of the total gene list. This is in contrast with the NMDA-RC gene set where multiple genes were involved in the enrichment signal in both CAGES and in BATS, consistent with the notion that it is variation in the network and not in a single gene, which contributes to normal variation in fluid ability.

In summary, large-scale molecular studies indicate that intelligence is polygenic^3,4^ that is compatible with a range of genetic models, the most extreme of which would be that all genes matter with roughly equal effect. Here, using GSEA, we tested the hypothesis that that some genes matter more than others. Specifically, we found that genes in pathways related to postsynaptic functioning are enriched. The results suggested that a major component of the postsynaptic region, the NMDA-RC, is preferentially associated with normal variation in intelligence. The NMDA-RC pathway appears to be specifically enriched for association with fluid ability, providing a lead towards understanding a source of some of the variation in human intelligence differences.

## Figures and Tables

**Table 1:** Shows the results of enrichment analysis on six candidate gene lists from the PSD in *gf* in the CAGES cohorts

*Complex name*	*Number of genes*	*Empirical* P*-value*	*FDR*
hPSD full	1386	0.628	0.705
hPSD consensus	714	0.242	0.542
NMDA-RC	181	0.002	0.221
mGlu5-RC	50	0.133	0.203
AMPA-RC	7	0.595	0.804
ARC	25	0.870	0.870
Replication samples			
NMDA-RC (BATS)	180	0.012	0.012
NMDA-RC (NCNG)	180	0.371	0.371

Abbreviations: AMPA-RC, α-amino-3-hydroxy-5-methyl-4-isoxazolepropionic acid receptor complex; BATS, Brisbane Adolescent Twin Study; FDR, false discovery rate; hPSD,human postsynaptic density; mGlu5-RC, the metabotropic glutamate receptor complex 5; NMDA-RC,

*N*-methyl-D-aspartate receptor signalling complex/membrane-associated guanylate kinase associated signalling complex; NCNG, Norwegian Cognitive NeuroGenetics. The replication of the NRSC gene set in both BATS and NCNG cohorts is included.
